# Psychological Stress and Anxiety in Autoimmune Diseases: A Cross-Sectional Case–Control Study

**DOI:** 10.3390/healthcare14162584

**Published:** 2026-08-17

**Authors:** Lucia Bubulac, Eliza Seche, Mirela Zivari, Constantin Erena, Dana-Maria Popescu-Spineni, Claudia Florina Bogdan-Andreescu, Cristina-Crenguța Albu, Tudor Georgescu, Dan Alexandru Slăvescu, Adrian Bobu

**Affiliations:** 1 Department of Family Medicine, Faculty of Medicine, “Carol Davila” University of Medicine and Pharmacy, 020021 Bucharest, Romania; lucia.bubulac@umfcd.ro; 2 Department of Dentistry, Faculty of Medicine and Pharmacy, University of Oradea, 410073 Oradea, Romania; secheeliza@uoradea.ro (E.S.); slavescudan@uoradea.ro (D.A.S.); 3 Department of Psychology and Educational Sciences, Faculty of Psychology, Ecological University of Bucharest, 061341 Bucharest, Romania; mirela.zivari@ueb.education; 4 Department of Psychology, Emergency University Hospital, 050098 Bucharest, Romania; 5 Internal Medicine Department, Bucharest Emergency Clinical Hospital, Faculty of Medicine, “Carol Davila” University of Medicine and Pharmacy, 020021 Bucharest, Romania; constantin.erena@umfcd.ro; 6 Department of Social Medicine, Faculty of Midwifery and Nursing, “Carol Davila” University of Medicine and Pharmacy, 020021 Bucharest, Romania; spineni.popescu@umfcd.ro; 7 Department of Biomedical Anthropology, “Francisc Reiner” Institute of Anthropology of the Romanian Academy, 050711 Bucharest, Romania; 8 Department of Specialty Disciplines, “Titu Maiorescu” University, 031593 Bucharest, Romania; claudia.andreescu@prof.utm.ro; 9 Department of Genetics, Faculty of Dentistry, “Carol Davila” University of Medicine and Pharmacy, 020021 Bucharest, Romania; 10 Doctoral School, Faculty of Medicine and Pharmacy, University of Oradea, 410073 Oradea, Romania; 11 Faculty of Automatic Control and Computers, National University of Science and Technology POLITEHNICA, 060042 Bucharest, Romania; adrian.bobu@stud.acs.upb.ro

**Keywords:** autoimmune diseases, psychological stress, state anxiety, trait anxiety, psychoneuroimmunology, biopsychosocial model, psychological screening

## Abstract

**Background:** Psychological distress has been frequently reported in individual autoimmune diseases, but evidence regarding shared psychological characteristics across heterogeneous autoimmune conditions remains limited. **Objective:** This study compared stress vulnerability, exposure to major life events, state and trait anxiety, and Type A behavioral characteristics between adults with autoimmune diseases and controls. **Methods:** This cross-sectional case–control study included 389 adults: 204 patients with autoimmune diseases and 185 controls. Participants completed the Stress Vulnerability Scale, Holmes–Rahe Life Stress Inventory, State–Trait Anxiety Inventory, and Jenkins Activity Survey. Between-group differences, effect sizes, and correlations among psychometric variables were examined. **Results:** Patients with autoimmune diseases had significantly higher stress vulnerability scores (Cohen’s d = 0.39) and Holmes–Rahe life-event burden (d = 0.54) than controls. State anxiety (d = 0.52) and trait anxiety (d = 0.68) were also higher (all *p* < 0.001). Type A behavioral characteristics did not differ significantly between groups. State and trait anxiety showed the strongest correlation (r = 0.755, *p* < 0.001). Disease duration was positively associated with Holmes–Rahe scores but not with stress vulnerability or anxiety. **Conclusions:** Adults with autoimmune diseases showed a higher psychological burden across several complementary psychometric dimensions. These findings support consideration of psychological screening within multidisciplinary autoimmune disease care. Longitudinal and disease-specific studies integrating biological, psychological, and social factors are required to clarify temporal and potentially bidirectional relationships.

## 1. Introduction

Autoimmune diseases (ADs) comprise a heterogeneous group of chronic disorders characterized by loss of immunological self-tolerance, aberrant activation of innate and adaptive immune responses, and progressive tissue and organ damage [1]. Beyond their organ-specific manifestations, autoimmune diseases exert a substantial impact on quality of life, emotional well-being, and psychological functioning [2]. In the present study, particular attention is directed toward two major psychological dimensions frequently associated with chronic disease burden: stress vulnerability and anxiety.

The pathogenesis of autoimmune diseases is increasingly recognized as a multifactorial process resulting from interactions between genetic susceptibility, environmental exposures, and psychosocial influences [3,4]. More than eighty autoimmune conditions have been identified, including rheumatoid arthritis, systemic lupus erythematosus, psoriasis, inflammatory bowel disease, autoimmune thyroiditis, type 1 diabetes mellitus, and multiple sclerosis [5]. Although these disorders present distinct clinical phenotypes, they share common immunopathogenic mechanisms, including dysregulated cytokine signaling, impaired regulatory T-cell activity, altered antigen presentation, and breakdown of central or peripheral immune tolerance [5].

Sex-related differences contribute significantly to autoimmune disease susceptibility and clinical expression. Diseases such as systemic lupus erythematosus and Sjögren’s syndrome predominantly affect women, reflecting the influence of sex chromosomes, X-chromosome inactivation patterns, sex-biased microRNAs, and hormonal factors on immune regulation [6]. Importantly, sex differences may also influence psychological responses to chronic disease and therefore represent a relevant confounding factor in studies evaluating stress and anxiety among autoimmune patients.

Although genetic factors contribute substantially to disease susceptibility, genome-wide association studies indicate that inherited genetic variation explains only part of disease heritability [7,8,9]. Environmental exposures, including infections, diet, pollutants, lifestyle factors, and psychosocial stress, interact with genetic predisposition through epigenetic and immunoregulatory mechanisms, influencing disease onset and progression [10,11].

Early holistic conceptions of health anticipated the contemporary integration of psychological, neuroendocrine, and immune processes within psychoneuroimmunology and systems medicine [12,13].

The classical biomedical model, focused primarily on autoantibodies and inflammatory cascades, cannot fully explain fluctuations in disease activity, symptom severity, or inter-individual variability in disease progression [14]. Similar interactions between psychological stress, anxiety, and disease evolution have been reported in other chronic disorders, including arterial hypertension and type 2 diabetes mellitus [15,16]. These observations support a biopsychosocial perspective integrating biological, psychological, and social determinants of health.

Within this biopsychosocial perspective, the psychological impact of autoimmune disease may also be influenced by the social context in which patients experience and manage chronic illness. Social factors may influence exposure to stressors, coping resources, anxiety, treatment adherence, and quality of life. Relevant factors include employment, financial strain, family responsibilities, healthcare access, social support, social isolation, stigma, and limitations in occupational or community participation. Accordingly, psychological distress in autoimmune disease should not be interpreted independently of the patient’s broader social environment.

Among these factors, psychological stress has emerged as an important contributor to immune dysregulation. Psychoneuroimmunology (PNI) research has shown that chronic stress can alter hypothalamic–pituitary–adrenal (HPA) axis activity and cortisol secretion and can contribute to glucocorticoid resistance [17,18,19,20,21]. These mechanisms are frequently observed in autoimmune diseases. Emotional dysregulation and chronic stress have also been associated with elevated inflammatory biomarkers, including interleukin-6 (IL-6), tumor necrosis factor-alpha (TNF-α), and C-reactive protein (CRP) [22]. These biomarkers may contribute to disease activity and symptom burden in chronic inflammatory conditions.

Anxiety represents another important component of the psychoneuroimmunological framework. Increased anxiety levels have been associated with heightened sympathetic nervous system activity, oxidative stress, and enhanced inflammatory signaling [23,24,25]. In autoimmune diseases, anxiety has been linked to increased fatigue, greater pain perception, reduced quality of life, and higher overall disease burden [23,24]. Although these associations do not establish causality, they support the relevance of psychological factors within the complex interactions between immune regulation and chronic disease.

The COVID-19 pandemic further illustrated the potential interactions among widespread psychological stress, anxiety, and immune regulation, particularly in susceptible populations [26,27,28,29,30,31].

Recent evidence indicates that peripheral inflammation and immune dysregulation may influence central nervous system function through cytokine-mediated signaling [32,33,34,35,36,37,38,39,40,41,42,43]. Neuroendocrine and gut–brain pathways may also contribute to interactions between stress responsiveness and immune regulation. Although these mechanisms were not directly assessed in the present study, they provide biological context for the investigated psychometric associations.

The global burden of autoimmune diseases has increased substantially over recent decades, with epidemiological studies estimating that up to 7–10% of the population may be affected by at least one autoimmune condition [44,45,46,47]. During the same period, population-based surveys have documented increasing levels of perceived psychological stress [48,49]. While these trends do not imply causal relationships, they support continued investigation of potential associations between stress, anxiety, immune regulation, and autoimmune disease outcomes.

Previous studies have documented elevated stress and anxiety in individual autoimmune conditions, including rheumatoid arthritis, systemic lupus erythematosus, autoimmune thyroiditis, psoriasis, and multiple sclerosis [2,23,24,32]. However, differences in study populations, assessment instruments, and analytical approaches limit comparisons across diseases. It therefore remains unclear whether stress vulnerability, exposure to major life events, and state and trait anxiety constitute shared psychological characteristics across heterogeneous autoimmune conditions.

A further limitation of the available evidence is that stress exposure, stress vulnerability, anxiety, and behavioral characteristics are frequently investigated separately. Their simultaneous assessment may provide a more integrated characterization of psychological burden. Nevertheless, autoimmune diseases differ substantially in clinical manifestations, severity, treatment, and prognosis. Findings from a heterogeneous cohort should therefore be interpreted as exploratory evidence of shared patterns rather than as proof of a universal psychological profile.

The present study compared four complementary psychological domains—stress vulnerability, exposure to major life events, state and trait anxiety, and Type A behavioral characteristics—between adults with autoimmune diseases and controls. A secondary objective was to examine the relationships among these dimensions and their associations with disease duration. The scientific contribution of the study lies in the simultaneous evaluation of these complementary psychometric dimensions using a common assessment protocol in a clinically heterogeneous autoimmune cohort.

### Theoretical Framework and Study Hypotheses

The study was guided by complementary biopsychosocial and psychoneuroimmunological perspectives. The biopsychosocial model conceptualizes health as the result of interacting biological, psychological, and social determinants. Within this framework, autoimmune disease may contribute to psychological distress through persistent symptoms, treatment burden, uncertainty, functional limitations, and changes in social participation. Conversely, chronic psychological stress may influence neuroendocrine and immune regulation through the hypothalamic–pituitary–adrenal axis, autonomic nervous system, and inflammatory signaling pathways. These relationships are potentially bidirectional; therefore, the present cross-sectional study examines associations and does not assume a causal direction.

Stress vulnerability reflects the individual tendency to experience adverse responses when coping demands exceed perceived adaptive resources. The Holmes–Rahe score reflects cumulative exposure to major life events during the preceding 12 months. State and trait anxiety distinguish transient anxiety from a relatively stable anxiety disposition. Type A behavioral characteristics were included to determine whether the observed psychological differences could be attributed to a broader behavioral pattern involving time urgency, competitiveness, and hostility.

Based on the biopsychosocial and psychoneuroimmunological framework and the empirical evidence summarized above regarding stress, anxiety, and psychological burden in autoimmune and chronic inflammatory conditions [17,18,19,20,21,22,23,24,32], the following hypotheses were examined:

**H1.**  *Participants with autoimmune diseases have higher stress vulnerability scores than controls.*

**H2.**  *Participants with autoimmune diseases have higher Holmes–Rahe life-event scores than controls.*

**H3.**  *Participants with autoimmune diseases have higher state- and trait-anxiety scores than controls.*

**H4.**  *Stress vulnerability, Holmes–Rahe life-event burden, and anxiety scores are positively correlated.*

**H5.**  *The prevalence of the Type A behavioral pattern differs between participants with autoimmune diseases and controls.*

## 2. Materials and Methods

### 2.1. Study Design

This cross-sectional case–control study evaluated stress vulnerability, cumulative life stress exposure, anxiety levels, and Type A behavioral characteristics. Patients with autoimmune diseases were compared with healthy controls. A translational clinical approach was adopted, focusing on psychological characteristics associated with autoimmunity as a generalized state of immune dysregulation rather than disease-specific manifestations.

To support translational implementation, questionnaire-derived psychometric and clinical data were digitally integrated into a structured electronic framework designed to facilitate standardized data collection, storage, processing, and statistical analysis.

### 2.2. Study Population

A total of 389 participants were enrolled in the study, including 204 patients diagnosed with autoimmune diseases and 185 healthy controls. Participants in the autoimmune disease group were analyzed collectively as a heterogeneous cohort in order to investigate psychological characteristics potentially associated with autoimmunity. This approach was chosen to explore common psychometric patterns across autoimmune diseases sharing underlying immunological, neuroendocrine, and inflammatory mechanisms. The distribution of autoimmune disease diagnoses within the patient cohort is presented in Table 1.

The study was academically coordinated through the Department of Family Medicine, Faculty of Medicine, “Carol Davila” University of Medicine and Pharmacy, Bucharest, Romania. Participant recruitment and psychometric assessments were conducted between September and December 2025 at the collaborating private Family Medicine Practice of Dr Lucia Bubulac in Bucharest, Romania. Patients with autoimmune diseases were identified through the Family Medicine practice and referrals from collaborating clinical services, whereas control participants were recruited through the same Family Medicine practice and community outreach. All participants provided written informed consent before enrollment and before the completion of any study-specific assessment.

#### 2.2.1. Inclusion Criteria

Participants were eligible for inclusion if they met the following criteria:

Autoimmune disease group:

- Age ≥ 18 years;
- Confirmed diagnosis of an autoimmune disease (established by a specialist physician based on clinical evaluation and relevant laboratory and immunological criteria);
- Stable clinical status, defined as the absence of acute disease exacerbation requiring hospitalization and the absence of major treatment modification at the time of evaluation; formal remission criteria or disease activity scores were not used as inclusion criteria;
- Ability to understand and independently complete all study questionnaires;
- Provision of written informed consent.

Control group:

- Age ≥ 18 years;
- Absence of diagnosed autoimmune, inflammatory, or psychiatric disorders;
- No current treatment with psychotropic medication;
- Ability to complete all assessments independently and provision of written informed consent.

#### 2.2.2. Exclusion Criteria

Participants from both groups were excluded if they:

- Had a history of severe psychiatric disorders (e.g., psychotic disorders or bipolar disorder);
- Were undergoing active psychiatric treatment at the time of assessment;
- Presented acute infectious or inflammatory conditions;
- Had cognitive impairment or any condition limiting comprehension or completion of the questionnaires.

### 2.3. Sociodemographic and Clinical Data Collection

Sociodemographic data were collected using a structured questionnaire and included age, sex, and area of residence (urban or rural). Clinical information regarding autoimmune disease diagnosis was obtained from medical documentation available to participants and recorded during the study assessment.

A total of 389 participants were included in the final analysis, comprising 204 patients with autoimmune diseases and 185 healthy controls. The autoimmune disease group showed a marked predominance of female participants (88.2%), whereas females accounted for 69.2% of the control group. In both groups, most participants originated from urban areas. Patients with autoimmune diseases were predominantly represented in the 30–49-year age interval, whereas the control group included a higher proportion of younger individuals aged 18–29 years. Available sex and area-of-residence characteristics are summarized in Table 2.

To provide a more comprehensive overview of the dataset while preserving participant confidentiality, all clinical, sociodemographic, and psychometric data are presented in aggregate form. The final analytical sample comprised 389 participants, including 204 patients with autoimmune diseases and 185 controls. The distribution of autoimmune disease diagnoses is presented in Table 1, while the available sociodemographic characteristics are summarized in Table 2. Complete psychometric outcome data were available for the principal between-group comparisons. Information on disease duration was available for 186 of the 204 patients with autoimmune diseases (91.2%). Disease-duration information was unavailable or insufficiently documented for the remaining 18 patients (8.8%); consequently, these participants were excluded only from analyses involving years since diagnosis. No directly identifying or individual-level participant information is reported.

### 2.4. Psychological Assessment

All participants completed a standardized battery of psychometric instruments administered under identical conditions. The instruments were selected to assess complementary dimensions of psychological burden rather than interchangeable constructs. The Stress Vulnerability Scale assessed individual susceptibility to stress in relation to lifestyle, health behaviors, coping resources, and perceived social support. The Holmes–Rahe Life Stress Inventory quantified cumulative exposure to major life events. The State–Trait Anxiety Inventory differentiated transient anxiety from a relatively stable anxiety disposition. The Jenkins Activity Survey evaluated Type A behavioral characteristics that could contribute to differences in stress-related outcomes. Together, these instruments provided a multidimensional assessment of stress exposure, stress vulnerability, anxiety, and behavioral characteristics.

The questionnaires were administered in Romanian using previously available Romanian-language versions. No instrument was translated specifically for the present study. The established item wording, response options, and scoring instructions of the Romanian-language versions were retained without modification. Questionnaire administration was supervised by trained study personnel, who provided standardized instructions and clarified only procedural aspects, without interpreting individual items or influencing participant responses.

#### 2.4.1. Stress Vulnerability Scale

Vulnerability to psychological stress was assessed using the 20-item Stress Vulnerability Scale (SVS) developed by Miller and Smith [50]. The instrument was selected because it evaluates lifestyle-related and psychosocial factors that may reduce an individual’s resistance to stress. These include health behaviors, sleep, physical activity, stimulant use, time management, leisure, and perceived social support.

The scale consists of 20 items rated on a five-point frequency scale ranging from 1 (“almost always”) to 5 (“never”), according to how often each statement applies to the respondent. All items are scored in the same direction; therefore, no reverse-scoring is required. The raw item scores are summed, producing a value between 20 and 100, after which 20 points are subtracted in accordance with the standard scoring procedure. The resulting adjusted total score ranges from 0 to 80, with higher scores indicating greater vulnerability to stress and lower scores indicating greater resistance to stress.

The Romanian-language version was administered using the same item structure and scoring procedure as the original 20-item instrument. The scale has previously been used in Romanian populations [51]. In the present study, the total score was analyzed primarily as a continuous variable.

#### 2.4.2. Holmes–Rahe Life Stress Inventory

Cumulative exposure to major life events was assessed using the original 43-item Holmes–Rahe Social Readjustment Rating Scale, also known as the Holmes–Rahe Life Stress Inventory [52]. This instrument was selected because it quantifies the accumulated burden of significant life changes. It complements the Stress Vulnerability Scale, which evaluates individual vulnerability to stress rather than exposure to stressful events.

The instrument includes 43 life events, each assigned a predefined Life Change Unit (LCU) weight according to the magnitude of readjustment typically associated with that event. Participants indicated which events they had experienced during the preceding 12 months, and the corresponding LCU weights were summed to obtain a total score. Higher total scores indicate greater cumulative exposure to major life changes. For descriptive interpretation, total scores were categorized as low (<150 LCU), moderate (150–299 LCU), or high (≥300 LCU) life-change burden. These categories follow the original scoring framework proposed by Holmes and Rahe [52].

The Holmes–Rahe score was analyzed primarily as a continuous variable, while the categories were used only for descriptive purposes. The instrument quantifies exposure to potentially stressful life changes and should not be interpreted as a diagnostic measure of perceived stress, psychiatric illness, or individual causation of physical disease.

#### 2.4.3. State–Trait Anxiety Inventory (STAI)

Anxiety was assessed using the 40-item adult form of the State–Trait Anxiety Inventory (STAI), comprising two separate 20-item subscales designed to distinguish between state anxiety and trait anxiety [53,54]. The STAI was selected because it permits the separate assessment of transient anxiety experienced at the time of evaluation and a relatively stable tendency to perceive situations as threatening.

The STAI-State subscale comprises 20 items evaluating how participants feel “at this moment,” whereas the STAI-Trait subscale comprises 20 items evaluating how participants generally feel. Each item is rated on a four-point Likert scale. In accordance with the standardized scoring procedure, positively worded items were reverse-scored before calculation of the subscale totals. Scores for each subscale range from 20 to 80, with higher scores indicating greater state or trait anxiety.

A previously available Romanian-language version was administered using the established item wording, response format, and standardized scoring instructions. The STAI has demonstrated good internal consistency and construct validity in adult populations [53,54]. In the present sample, both the STAI-State and STAI-Trait subscales demonstrated good internal consistency, with Cronbach’s α values exceeding 0.80.

STAI-State and STAI-Trait scores were analyzed primarily as continuous variables. The scores were interpreted as indicators of anxiety severity and were not used independently to establish a clinical diagnosis of an anxiety disorder.

#### 2.4.4. Jenkins Activity Survey

Type A behavioral characteristics were evaluated using the adult Form C of the Jenkins Activity Survey (JAS). The instrument was included to examine whether between-group differences in stress and anxiety could be related to a broader behavioral pattern characterized by time urgency, competitiveness, achievement orientation, and hostility [55].

The JAS Form C is a 52-item self-administered multiple-choice questionnaire. Its scoring system generates a standardized global Type A score from 21 weighted items embedded within the complete questionnaire. It also provides component scores for speed and impatience, job involvement, and hard-driving competitiveness. Responses were scored using the standardized weighted scoring algorithm described by Jenkins et al. [55]. The resulting global Type A score is standardized to a mean of 0 and a standard deviation of 10. Scores greater than 0 indicate a behavioral pattern oriented toward Type A characteristics, whereas scores of 0 or lower indicate a pattern oriented toward Type B characteristics. For the categorical analysis conducted in the present study, participants with global scores greater than 0 were classified as presenting a Type A behavioral pattern. Higher positive scores indicate a stronger expression of Type A behavioral characteristics.

A previously available Romanian-language version was administered using the established item structure, response options, and scoring procedure. No translation or modification of the instrument was performed specifically for the present study. Previous validation research has reported adequate internal consistency for the global Type A scale, with a reliability coefficient of approximately 0.85 [55]. In the present study, the JAS was used primarily for the standardized categorical classification of participants as presenting or not presenting a Type A behavioral pattern. The instrument was used for behavioral characterization and not as a diagnostic measure of a personality disorder or cardiovascular risk.

#### 2.4.5. Reliability and Measurement Quality

Internal consistency was evaluated using Cronbach’s α for the multi-item instruments designed to assess a common underlying construct. Values of at least 0.70 were considered acceptable for group-level comparisons. In the present sample, the Stress Vulnerability Scale demonstrated acceptable internal consistency (Cronbach’s α > 0.75), while both State–Trait Anxiety Inventory subscales demonstrated good internal consistency (Cronbach’s α > 0.80). These findings support the reliability of the SVS and STAI scores in the study population.

Cronbach’s α was not calculated for the Holmes–Rahe Life Stress Inventory because it is a weighted checklist of discrete life events rather than a reflective unidimensional scale. The occurrence of one life event is not necessarily expected to correlate with the occurrence of another; therefore, internal consistency is not an appropriate reliability criterion for this instrument.

Questionnaire responses were reviewed for completeness immediately after administration. Scores were calculated according to the standardized instructions for each instrument. This included reverse-scoring the relevant STAI items and applying the standardized weighted scoring procedures for the Holmes–Rahe inventory and Jenkins Activity Survey. No item-level imputation was performed. If the responses required to calculate a scale or subscale score were incomplete, the corresponding score was treated as missing and excluded from analyses involving that measure.

Evidence of measurement validity was based on the original development and validation studies corresponding to the instruments administered. No new psychometric instrument was developed or adapted in the present study.

### 2.5. Data Collection Procedure

Psychometric assessments were conducted between September and December 2025 at the private Family Medicine Practice of Dr Lucia Bubulac, Bucharest, Romania. The study was academically coordinated through the Department of Family Medicine, Faculty of Medicine, “Carol Davila” University of Medicine and Pharmacy, Bucharest, Romania. It was implemented at the collaborating private practice in accordance with the research protocol approved by the university Ethics Committee.

Participants completed the questionnaires individually in a quiet consultation room under standardized conditions and without a fixed time limit. Before questionnaire completion, trained study personnel provided the same standardized written and verbal instructions to all participants. Study personnel clarified only procedural aspects and did not interpret individual items, suggest responses, or otherwise influence participants’ answers.

Questionnaires were reviewed immediately after completion to identify inadvertently unanswered items. When an item had been left unanswered, the questionnaire was returned to the participant only if the participant was willing to reconsider the omission. Participants were not required to answer any question they preferred to leave unanswered, and investigators did not infer, supply, or modify responses on their behalf. The missing-data and score-calculation procedures are described in Section 2.4.5.

Questionnaire responses were subsequently entered into a customized electronic data-management framework using predefined variable names, coding conventions, permissible score ranges, and automated completeness and range checks. Values identified as missing, inconsistent, or outside the theoretically permissible range were verified against the original source questionnaire. Corrections were made only when a data-entry discrepancy could be confirmed from the original questionnaire; original participant responses were not altered. The dataset used for statistical analysis was pseudonymized, and access to the source documents and electronic database was restricted to authorized members of the research team.

### 2.6. Statistical Analysis

Statistical analyses were performed using two complementary analytical approaches:

1. IBM SPSS Statistics, version 30.0 (IBM Corp., Armonk, NY, USA), which was used for the primary statistical analyses.
2. A custom MATLAB-based analytical script (MathWorks R2024b, Natick, MA, USA), which was used to verify the statistical calculations and generate the computational visualizations.

Continuous variables were summarized as means and standard deviations, whereas categorical variables were reported as absolute frequencies and percentages. Distributional assumptions were evaluated by visual inspection of histograms and boxplots and by examining the data for extreme or potentially influential observations. No severe departures from normality were identified for the principal psychometric outcomes. The independence of observations was ensured by the study design.

Between-group comparisons of continuous psychometric outcomes were performed using independent-samples *t*-tests. This method was selected to compare the mean psychometric scores of two independent groups. Homogeneity of variance was evaluated using Levene’s test [56]. When the equal-variance assumption was not satisfied, Welch’s *t*-test and the corresponding adjusted degrees of freedom were used.

Results of the continuous between-group comparisons were reported as group-specific means and standard deviations, mean differences with 95% confidence intervals, t-statistics, degrees of freedom, and *p*-values. The magnitude of the between-group differences was quantified using Cohen’s d. Values of approximately 0.20, 0.50, and 0.80 were interpreted as small, moderate, and large standardized effects, respectively.

Associations between categorical variables were evaluated using Pearson’s chi-square test [57,58]. This test was used to compare the distribution of the Type A psychobehavioral pattern between the autoimmune disease and control groups.

Linear associations among psychometric scores, age, and disease duration were examined using Pearson’s correlation coefficient. Correlation coefficients were interpreted according to their direction and magnitude and were reported with the corresponding *p*-values.

Analyses were conducted using available cases for each outcome. No statistical imputation of missing data was performed. The number of observations included in each analysis is reported in the corresponding table or figure legend. All statistical tests were two-tailed, and a *p*-value below 0.05 was considered statistically significant. Exact p-values were reported, except when *p* < 0.001. Confidence intervals were calculated at the 95% level.

### 2.7. Data Visualization

To facilitate comprehensive interpretation of psychometric outcomes, multidimensional graphical representations were generated using MATLAB (MathWorks R2024b, Natick, MA, USA). These visualizations integrated score distributions, between-group comparisons, age-related trends, disease-duration associations, and individual data-point dispersion within unified graphical frameworks. Additional computational visualizations, including correlation heatmaps, pairwise scatterplot matrices, and comparative boxplots, were generated to support multivariate exploration of relationships among psychological variables.

### 2.8. Ethical Considerations

The study was conducted in accordance with the Declaration of Helsinki and was approved by the Ethics Committee of the “Carol Davila” University of Medicine and Pharmacy, Bucharest, Romania (Approval No. 20158; 12 August 2025). The study was academically coordinated through the Department of Family Medicine of the same university and implemented at the collaborating private Family Medicine practice. All participants provided written informed consent before enrollment and before the completion of any study-specific assessment.

## 3. Results

The results of the psychometric assessments and computational analyses are presented below. All 389 participants were included in the principal between-group psychometric analyses. Analyses involving disease duration were restricted to the 186 patients with valid information regarding the number of years since autoimmune disease diagnosis.

### 3.1. Statistical Results

#### 3.1.1. Vulnerability to Stress

Patients with autoimmune diseases exhibited significantly higher vulnerability to stress scores compared with healthy controls (26.31 ± 14.51 vs. 21.00 ± 12.58). The assumption of homogeneity of variances was satisfied (Levene’s test, *p* = 0.227).

Independent samples *t*-test revealed a statistically significant difference between groups (*t = 3.836, df = 387, *p* < 0.001), with a mean difference of 5.31 points (95% CI: 2.59–8.03). The magnitude of this difference corresponded to a small-to-moderate effect size (Cohen’s *d* = 0.39), indicating an increase in stress vulnerability among patients with autoimmune diseases.

**Figure 1:**
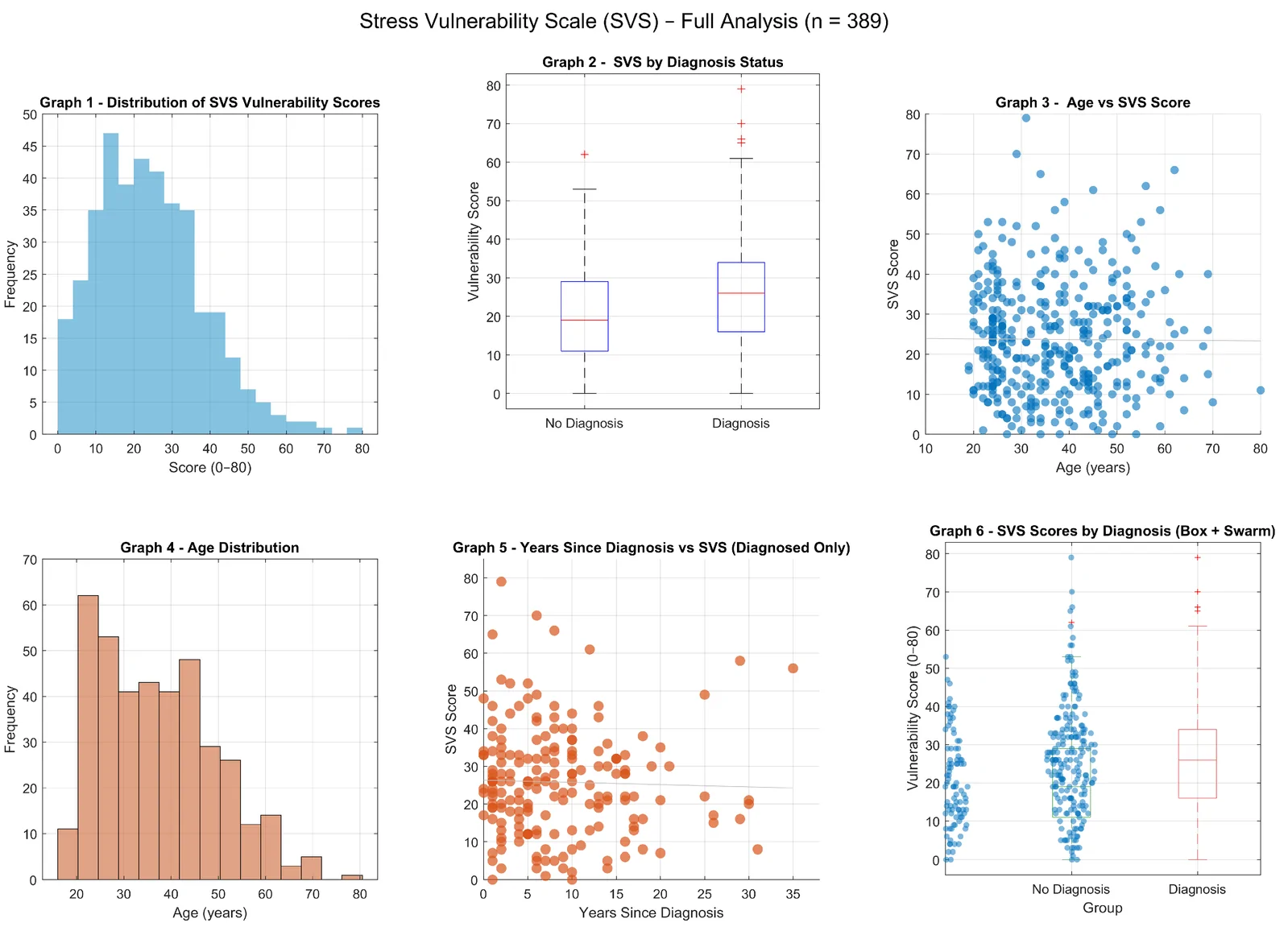
Integrated graphical analysis of Stress Vulnerability Scale (SVS) scores. Legend: (**Graph 1**) Histogram of SVS scores across the entire study population (*n* = 389), illustrating the distribution of stress vulnerability levels. (**Graph 2**) Boxplot comparing SVS scores between participants with and without autoimmune diseases, demonstrating higher median values and greater variability among affected individuals. (**Graph 3**) Scatterplot showing the relationship between age and SVS scores in the overall sample, including the fitted linear regression line. (**Graph 4**) Histogram of age distribution within the study population. (**Graph 5**) Scatterplot depicting the association between years since diagnosis and SVS scores among participants with autoimmune diseases (*n* = 186 with valid disease-duration data). (**Graph 6**) Combined boxplot and swarm plot illustrating individual score dispersion, central tendency, and between-group differences in stress vulnerability. Red plus signs (+) in (**Graph 2** and **Graph 6**) indicate outliers.

The results are summarized in Table 3 and graphically presented in Figure 1.

Figure 1 provides an integrated visualization of score distribution, between-group differences, and the associations between stress vulnerability, age, and disease duration.

The custom MATLAB-based analysis yielded results consistent with those obtained using SPSS, demonstrating significantly higher stress vulnerability scores among participants with autoimmune diseases. No significant association was observed between years since diagnosis and stress vulnerability scores (r = −0.030, *p* = 0.6865). This finding suggests that stress vulnerability may be more strongly related to current psychosocial burden and disease-related challenges than to disease duration itself.

#### 3.1.2. Cumulative Life Stress Exposure: Holmes–Rahe Stress Scale

Mean Holmes–Rahe Stress Scale scores were significantly higher in patients with autoimmune diseases compared with controls (339.25 ± 197.89 vs. 237.51 ± 174.54). Variance homogeneity was confirmed (Levene’s test, *p* = 0.065).

The between-group difference was highly significant (t = 5.354, df = 387, *p* < 0.001), with a mean difference of 101.73 points (95% CI: 64.37–139.09). This difference was associated with a moderate effect size (Cohen’s d = 0.54), reflecting substantially increased cumulative life stress exposure among participants with autoimmune diseases.

**Figure 2:**
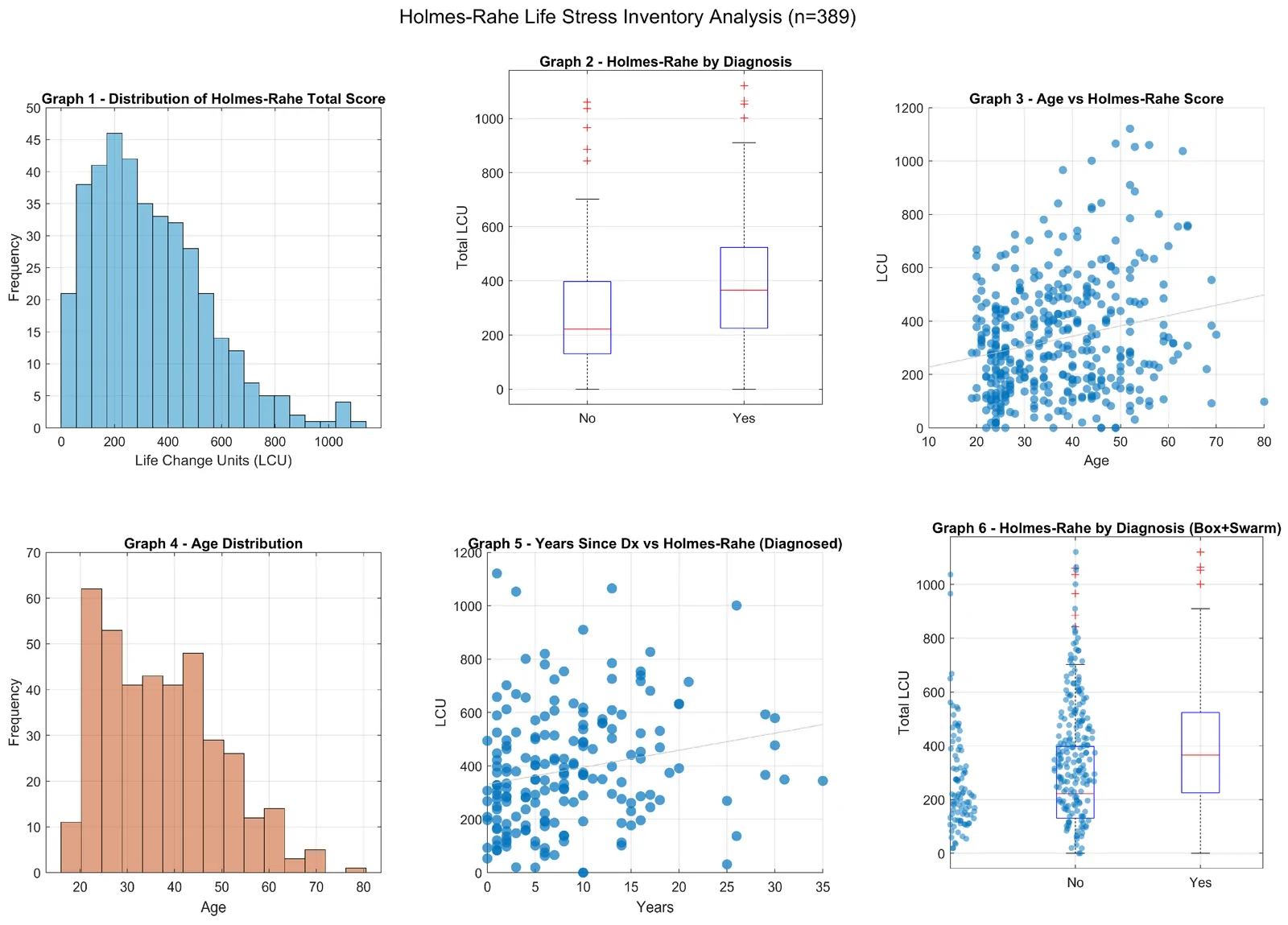
Integrated graphical analysis of Holmes–Rahe Life Stress Inventory scores. Legend: (**Graph 1**) Histogram showing the distribution of Holmes–Rahe total Life Change Unit (LCU) scores across the entire study population (*n* = 389). (**Graph 2**) Boxplot comparing Holmes–Rahe total scores between participants with and without autoimmune diseases, demonstrating higher cumulative stress exposure among affected individuals. (**Graph 3**) Scatterplot illustrating the relationship between age and Holmes–Rahe total scores in the overall sample, including the fitted linear regression line. (**Graph 4**) Histogram of age distribution within the study population. (**Graph 5**) Scatterplot depicting the association between years since diagnosis and Holmes–Rahe total scores among participants with autoimmune diseases (*n* = 186 with valid disease-duration data). (**Graph 6**) Combined boxplot and swarm plot illustrating individual score dispersion, central tendency, and between-group differences in cumulative life stress exposure. Red plus signs (+) in (**Graph 2** and **Graph 6**) indicate outliers. Blue points in (**Graph 3** and **Graph 5**) represent individual observations, and the solid lines represent the fitted linear regression lines.

The results are summarized in Table 4 and comprehensively visualized in Figure 2.

Figure 2 provides an integrated visualization of cumulative life stress exposure, between-group differences, and the associations between Holmes–Rahe scores, age, and disease duration.

The custom MATLAB-based analysis yielded results consistent with those obtained using SPSS, demonstrating significantly higher cumulative life stress exposure among participants with autoimmune diseases. The moderate effect size (Cohen’s d = 0.54) indicates a statistically significant between-group difference of moderate standardized magnitude. A modest but statistically significant positive correlation was observed between years since diagnosis and Holmes–Rahe total scores (r = 0.207, *p* = 0.0045). This finding suggests that longer disease duration may be associated with increased cumulative life stress exposure.

#### 3.1.3. State Anxiety (STAI-1)

State anxiety scores were significantly elevated in patients with autoimmune diseases compared with healthy controls (43.46 ± 12.62 vs. 36.91 ± 12.32). The assumption of equal variances was met (Levene’s test, *p* = 0.982).

Statistical analysis confirmed a significant between-group difference (t = 5.172, df = 387, *p* < 0.001), with a moderate effect size (Cohen’s d = 0.52). This result indicates higher acute anxiety levels among participants with autoimmune diseases.

**Figure 3:**
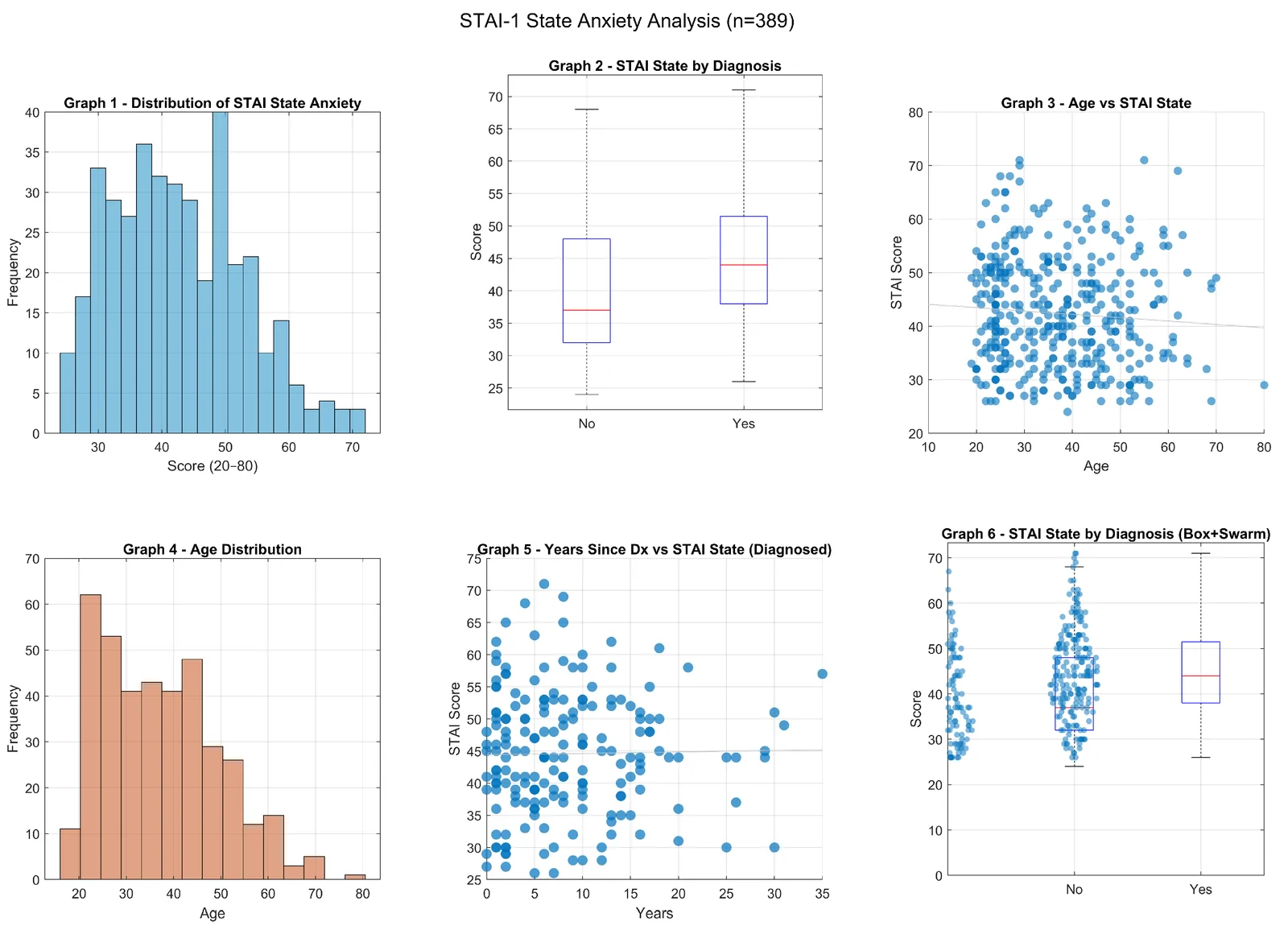
Integrated graphical analysis of State Anxiety Inventory (STAI-1) scores. Legend: (**Graph 1**) Histogram of STAI-1 scores across the entire study population (*n* = 389), illustrating the distribution of state anxiety levels. (**Graph 2**) Boxplot comparing STAI-1 scores between participants with and without autoimmune diseases, demonstrating higher anxiety levels among affected individuals. (**Graph 3**) Scatterplot showing the relationship between age and STAI-1 scores in the overall sample, including the fitted linear regression line. (**Graph 4**) Histogram of age distribution within the study population. (**Graph 5**) Scatterplot depicting the association between years since diagnosis and STAI-1 scores among participants with autoimmune diseases (*n* = 186 with valid disease-duration data). (**Graph 6**) Combined boxplot and swarm plot illustrating individual score dispersion, central tendency, and between-group differences in state anxiety. Blue points in (**Graph 3** and **Graph 5**) represent individual observations, and the solid lines represent the fitted linear regression lines.

The results are summarized in Table 5 and comprehensively visualized in Figure 3.

Figure 3 provides an integrated visualization of state anxiety distribution, between-group differences, and the associations between STAI-1 scores, age, and disease duration.

The custom MATLAB-based analysis yielded results consistent with those obtained using SPSS, demonstrating significantly higher state anxiety levels among participants with autoimmune diseases. The observed effect size (Cohen’s d = 0.52) indicates a statistically significant between-group difference of moderate standardized magnitude in state anxiety. No significant association was observed between years since diagnosis and STAI-1 scores (r = 0.017, *p* = 0.8221). This finding suggests that state anxiety may be more closely related to current disease-related experiences than to disease duration.

#### 3.1.4. Trait Anxiety (STAI-2)

Trait anxiety scores were also significantly higher in the autoimmune disease group (46.95 ± 10.29) compared with controls (39.49 ± 11.76). Due to unequal variances (Levene’s test, *p* = 0.004), the corrected *t*-test was applied.

The difference remained highly significant (t = 6.624, df = 367.6, *p* < 0.001), with a moderate-to-large effect size (Cohen’s d = 0.68). This finding suggests a persistent anxiety disposition associated with autoimmune disease status.

**Figure 4:**
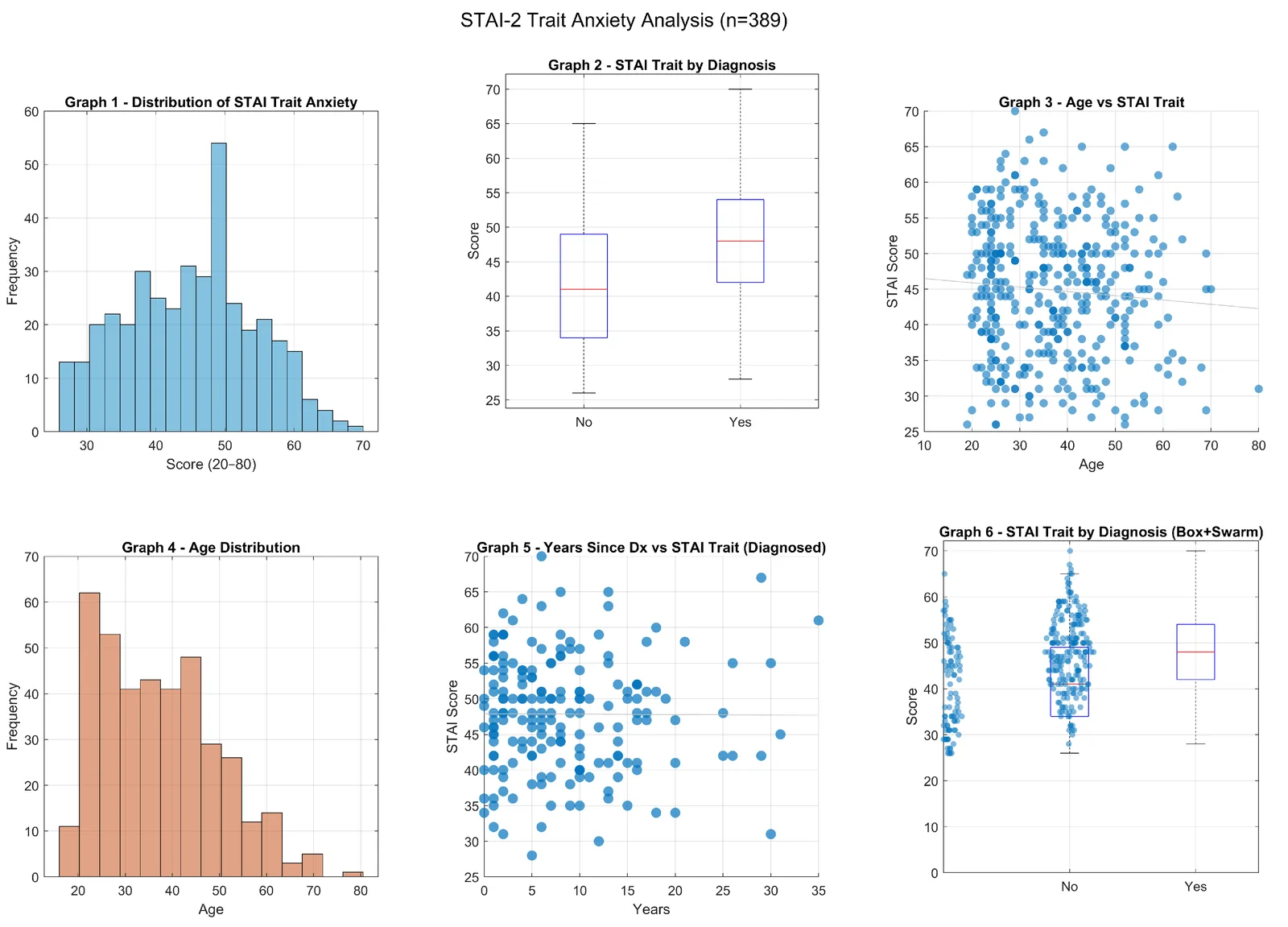
Integrated graphical analysis of Trait Anxiety Inventory (STAI-2) scores. Legend: (**Graph 1**) Histogram of STAI-2 scores across the entire study population *(n* = 389), illustrating the distribution of trait anxiety levels. (**Graph 2**) Boxplot comparing STAI-2 scores between participants with and without autoimmune diseases, demonstrating higher trait anxiety levels among affected individuals. (**Graph 3**) Scatterplot showing the relationship between age and STAI-2 scores in the overall sample, including the fitted linear regression line. (**Graph 4**) Histogram of age distribution within the study population. (**Graph 5**) Scatterplot depicting the association between years since diagnosis and STAI-2 scores among participants with autoimmune diseases (*n* = 186 with valid disease-duration data). (**Graph 6**) Combined boxplot and swarm plot illustrating individual score dispersion, central tendency, and between-group differences in trait anxiety. Blue points in (**Graph 3** and **Graph 5**) represent individual observations, and the solid lines represent the fitted linear regression lines.

The results are summarized in Table 5 and comprehensively visualized in Figure 4.

Figure 4 provides an integrated visualization of trait anxiety distribution, between-group differences, and the associations between STAI-2 scores, age, and disease duration.

The custom MATLAB-based analysis yielded results consistent with those obtained using SPSS, demonstrating significantly higher trait anxiety levels among participants with autoimmune diseases. The observed effect size (Cohen’s d = 0.68) indicates a moderate-to-large difference between groups, suggesting a stronger predisposition toward anxiety in individuals affected by autoimmune diseases. No significant association was observed between years since diagnosis and STAI-2 scores (r = −0.006, *p* = 0.9398), indicating that trait anxiety appears to be largely independent of disease duration.

#### 3.1.5. Type A Psycho-Behavioral Pattern

The prevalence of the Type A psycho-behavioral pattern was comparable between groups, being identified in 41.7% of patients with autoimmune diseases and 40.5% of healthy controls.

Chi-square analysis showed no statistically significant association between autoimmune disease status and Type A behavioral classification, *χ*^2^(1) = 0.051, *p* = 0.822, Cramér’s V = 0.011, indicating a negligible association. The distribution of the Type A psycho-behavioral pattern is presented in Supplementary Figure S1.

In contrast to stress vulnerability and anxiety, the distribution of the Type A psychobehavioral pattern did not differ significantly between autoimmune patients and healthy controls. This finding indicates that the observed psychological differences are not attributable to baseline personality structure characterized by competitiveness, hostility, or time urgency [59].

Because no statistically significant differences were identified between groups, Type A behavioral characteristics were not included in subsequent correlation analyses or in the discussion of the principal findings.

#### 3.1.6. Summary of Main Findings

In summary, patients with autoimmune diseases demonstrated significantly higher vulnerability to stress, cumulative life stress exposure, state anxiety, and trait anxiety compared with healthy controls. Effect sizes ranged from small-to-moderate for stress vulnerability (Cohen’s d = 0.39) to moderate-to-large for trait anxiety (Cohen’s d = 0.68). Overall, these effects indicate a substantial psychological burden associated with autoimmune disease status.

No significant associations were identified between disease duration and stress vulnerability, state anxiety, or trait anxiety. In contrast, cumulative life stress exposure showed a modest positive correlation with years since diagnosis, suggesting a potential increase in stress burden over the course of long-term disease management.

The custom MATLAB-based analytical framework confirmed all findings obtained through SPSS, demonstrating high consistency between the two analytical approaches and supporting the robustness and reproducibility of the results.

No statistically significant differences were observed regarding Type A behavioral characteristics between patients with autoimmune diseases and healthy controls.

## 4. Discussion

The present study provides consistent evidence that patients with autoimmune diseases exhibit significantly increased psychological stress vulnerability, cumulative life stress exposure, and anxiety compared with healthy controls. By adopting a translational clinical approach and analyzing autoimmune diseases as a heterogeneous group, the findings support the concept that psychological distress represents a common feature of autoimmunity rather than a disease-specific phenomenon.

In addition to conventional statistical analyses, the integration of psychometric data within a structured computational framework enabled multivariate exploration of the relationships among stress vulnerability, cumulative life stress burden, and anxiety dimensions. This approach facilitated a more comprehensive characterization of the psychological profile associated with autoimmune diseases and supports the future development of digitally assisted clinical monitoring strategies.

### 4.1. Psychological Stress Vulnerability and Autoimmunity

One of the key findings of this study is the significantly higher vulnerability to stress observed in patients with autoimmune diseases. Although the effect size was small-to-moderate, it was consistent and clinically relevant, indicating that autoimmune patients are more susceptible to stress-related psychological and somatic responses. This finding aligns with previous evidence suggesting that chronic stress can influence immune regulation through sustained activation of the hypothalamic–pituitary–adrenal axis and sympathetic nervous system pathways, ultimately promoting immune dysregulation [60].

Although the autoimmune cohort included clinically heterogeneous disorders, the consistent elevation of stress-related measures across the study population suggests the presence of shared psychological dimensions that may transcend disease-specific characteristics.

Importantly, stress vulnerability reflects not only exposure to stressors but also individual coping capacity. The increased vulnerability identified in this cohort suggests that autoimmune diseases may be associated with reduced stress resilience, potentially amplifying inflammatory responses and contributing to disease persistence or exacerbation [61].

Patients with autoimmune diseases also demonstrated significantly higher cumulative life stress exposure, as assessed by the Holmes–Rahe Stress Scale. The moderate effect size observed for this variable underscores the potential relevance of long-term stress accumulation in autoimmune pathology.

From a clinical perspective, the elevated Holmes–Rahe scores suggest that autoimmune patients may experience a sustained burden of stressors extending beyond transient psychological distress. Chronic exposure to major life stressors has been shown to affect immune homeostasis by altering cytokine profiles, glucocorticoid sensitivity, and inflammatory signaling pathways [62].

The significant positive association identified between disease duration and Holmes–Rahe scores suggests that cumulative stress burden may progressively increase during long-term disease management. This finding highlights the importance of considering chronic psychosocial stress as a potential contributor to disease burden and quality-of-life impairment in autoimmune populations.

Collectively, these findings support the concept that psychological stress and cumulative life stress exposure represent important components of the psychoneuroimmunological mechanisms involved in autoimmune diseases. Chronic stress may act both as a contributing factor and as a perpetuating influence within the complex interactions linking neuroendocrine regulation, immune function, and disease expression.

### 4.2. Anxiety as a Core Psychological Feature of Autoimmunity

Both state anxiety and trait anxiety were significantly elevated in patients with autoimmune diseases compared with healthy controls. Notably, the largest effect size observed in the present study corresponded to trait anxiety, indicating a stable anxiety disposition rather than a purely situational response. This finding suggests that anxiety may represent a central psychological dimension associated with autoimmune diseases [63].

Trait anxiety has been associated with altered autonomic balance, increased pro-inflammatory cytokine production, and dysregulation of stress-response systems. Elevated trait anxiety may therefore contribute to persistent psychological burden and interact with biological pathways involved in immune regulation. These findings support the conceptualization of anxiety as an important component of the psychoneuroimmunological framework of autoimmunity [64].

In contrast, elevated state anxiety likely reflects ongoing disease-related stressors, symptom burden, uncertainty regarding disease progression, and the challenges associated with long-term treatment and monitoring [65]. The absence of a significant association between disease duration and either anxiety measure suggests that anxiety levels may be more strongly influenced by current disease-related experiences and individual psychological characteristics than by the time elapsed since diagnosis.

From a clinical perspective, systemic inflammation may represent a shared biological pathway through which psychological stress and anxiety interact with autoimmune disease processes. Persistent inflammatory activity can exacerbate symptom burden, fatigue, and pain while contributing to neuropsychological manifestations. These observations are consistent with potential bidirectional interactions between psychological distress and immune dysregulation.

The marked predominance of female patients in the present autoimmune cohort may partly reflect sex-specific immunological and hormonal factors. Estrogen fluctuations, particularly during the menopausal transition, have been associated with heightened systemic inflammation and may influence susceptibility to autoimmune disease expression and anxiety-related symptoms [66,67].

Taken together, these findings indicate that anxiety represents a clinically relevant psychological characteristic of autoimmune disease populations and support the integration of psychological assessment and mental health interventions into comprehensive autoimmune disease management strategies.

### 4.3. Absence of Association with Type A Psychobehavioral Pattern

In contrast to stress vulnerability and anxiety, the distribution of the Type A psychobehavioral pattern did not differ significantly between autoimmune patients and healthy controls. This finding indicates that the observed psychological differences are not attributable to baseline personality structure characterized by competitiveness, hostility, or time urgency [59].

The absence of a between-group difference in Type A behavioral classification indicates that the observed differences in stress and anxiety were not explained by the measured Type A behavioral pattern. However, the cross-sectional design does not permit conclusions regarding the causal origin of psychological distress. Psychological distress may represent a potential target for assessment and supportive intervention; however, the effectiveness of such interventions was not evaluated in the present study and requires confirmation in prospective studies.

### 4.4. Computational Data Visualization and Correlation Analysis

To complement conventional statistical analyses, psychometric data were further processed using a custom MATLAB-based computational framework. The MATLAB environment enabled reproducible implementation of data preprocessing, score computation, correlation analysis, effect-size estimation, and multidimensional visualization through scripted analytical procedures.

The computational framework integrated the four psychometric dimensions investigated in this study—stress vulnerability (SVS), cumulative life stress burden (Holmes–Rahe), state anxiety (STAI-1), and trait anxiety (STAI-2)—into a unified analytical environment. This approach facilitated the exploration of interrelationships among variables and enabled visualization of complex associations that may not be readily apparent through conventional statistical reporting. A summary of descriptive statistics for all psychometric measures across the entire study population is presented in Supplementary Table S1.

To provide an integrated overview of the principal psychometric outcomes, between-group comparisons were summarized across all investigated dimensions (Table 6). Participants with autoimmune diseases consistently exhibited higher scores for stress vulnerability, cumulative life stress exposure, state anxiety, and trait anxiety compared with healthy controls. Moderate effect sizes were observed for Holmes–Rahe stress burden, state anxiety, and trait anxiety. Stress vulnerability showed a small-to-moderate effect size. Together, these findings support the presence of a substantial psychological burden associated with autoimmune disease status.

For clinical interpretability, psychometric scores were additionally categorized into low, moderate, and high levels according to instrument-specific cut-off values. The distribution of participants across these categories according to autoimmune disease status is presented in Supplementary Table S2.

**Figure 5:**
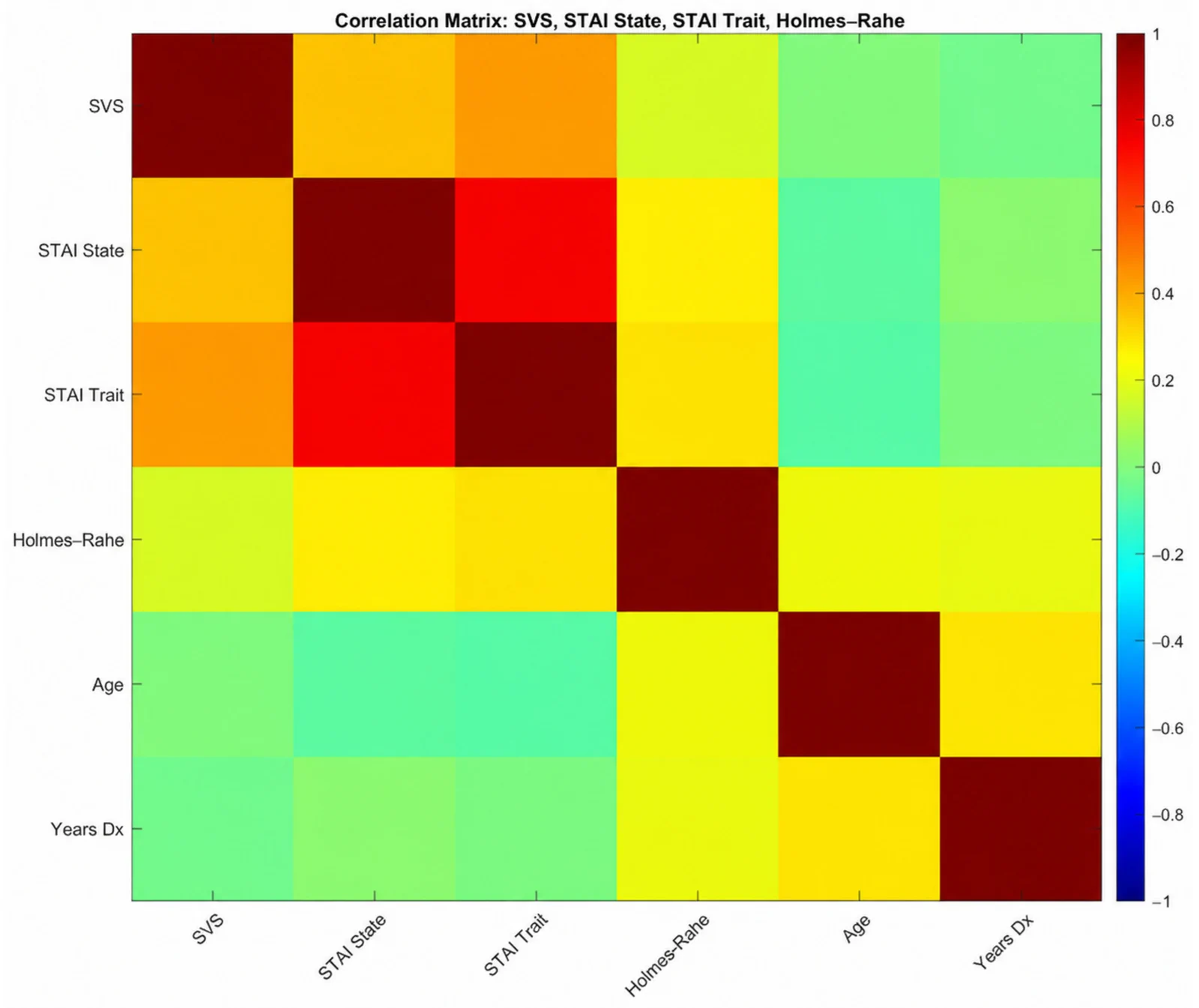
Correlation heatmap displaying interrelationships among SVS vulnerability, STAI State anxiety, STAI Trait anxiety, and Holmes–Rahe total score.

**Figure 6:**
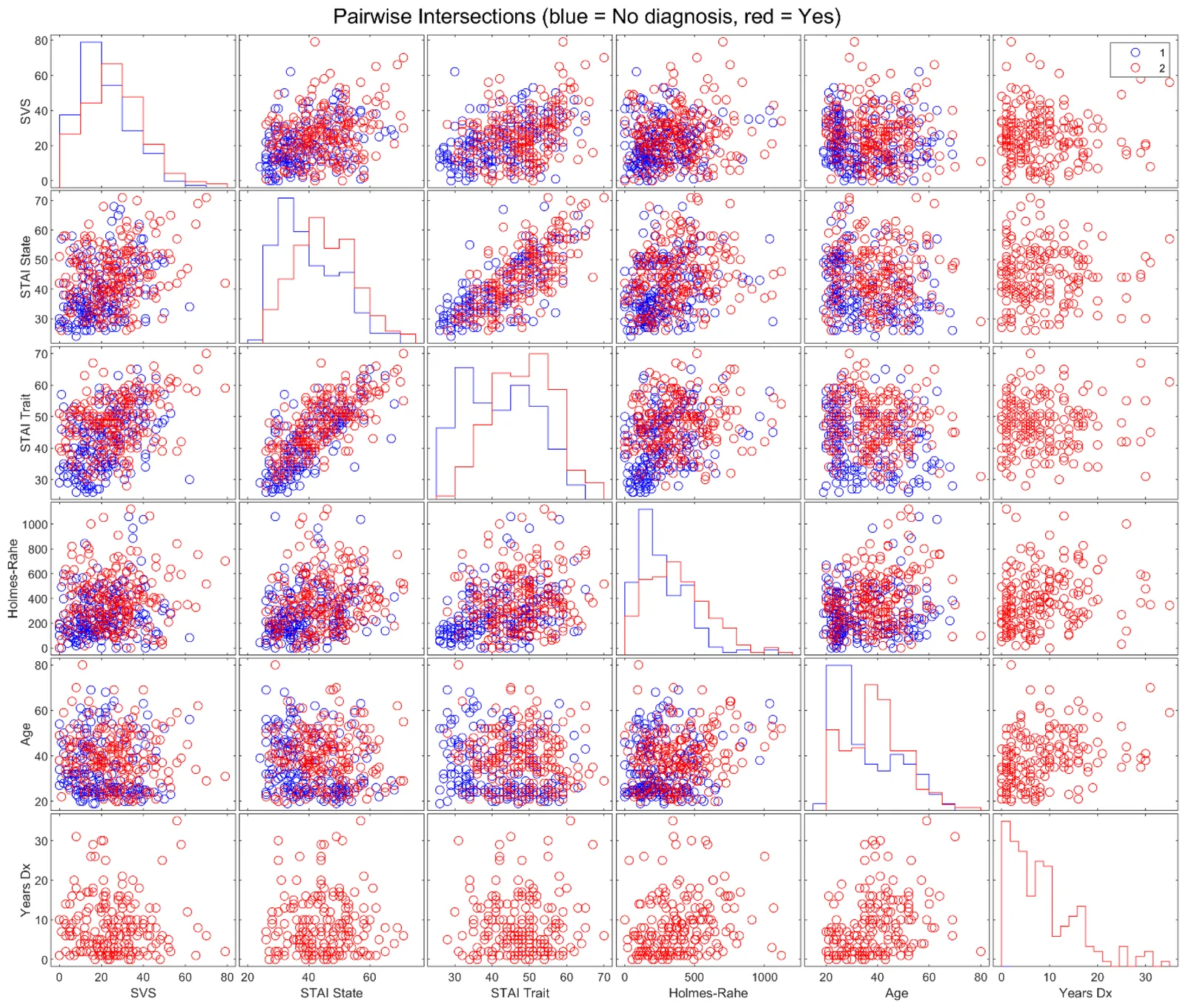
Pairwise scatterplot matrix showing relationships among the four psychometric measures according to diagnosis status. Blue open circles represent participants without autoimmune diseases, whereas red open circles represent participants with autoimmune diseases. In the diagonal panels, the blue and red stepped lines represent the corresponding group-specific frequency distributions, where applicable. Years since diagnosis is presented only for participants with autoimmune diseases.

**Figure 7:**
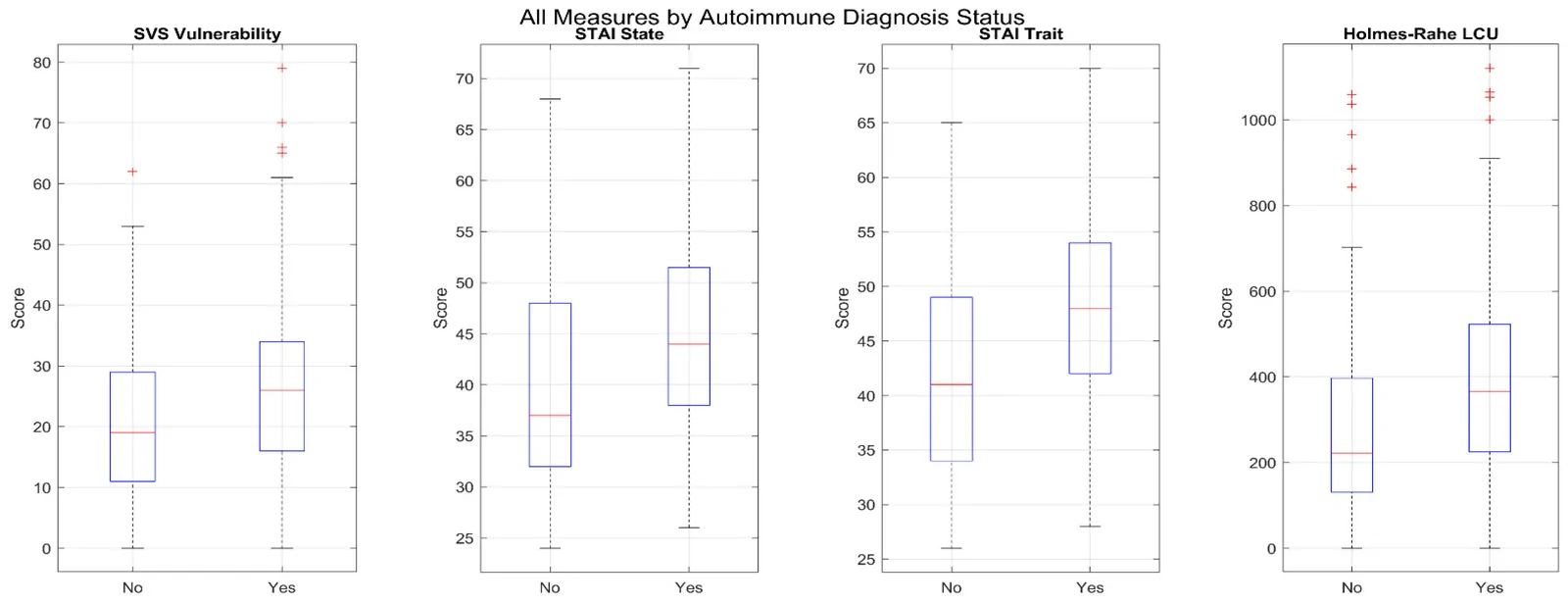
Comparative boxplots of SVS vulnerability, STAI State anxiety, STAI Trait anxiety, and Holmes–Rahe total scores in participants with and without autoimmune diseases.

The MATLAB-derived analyses confirmed the findings obtained using SPSS, supporting the robustness, reproducibility, and computational consistency of the analytical workflow. The resulting correlation structure among the investigated psychometric variables is illustrated in Figure 5, whereas multidimensional relationships and group-specific distributions are presented in Figure 6 and Figure 7.

Pearson correlation analyses revealed several significant interrelationships among the investigated psychometric variables, age, and disease duration (Table 7). The strongest positive association was observed between state and trait anxiety (r = 0.755, *p* < 0.001), while stress vulnerability demonstrated moderate positive correlations with both anxiety dimensions. Holmes–Rahe scores were positively associated with stress vulnerability, state anxiety, and trait anxiety, supporting the existence of interconnected psychological constructs within the study population. Additionally, Holmes–Rahe scores showed modest positive associations with age (r = 0.217, *p* < 0.001) and years since diagnosis (r = 0.207, *p* = 0.0045).

Notably, disease duration showed a significant positive association only with cumulative life stress exposure (Holmes–Rahe score), whereas no meaningful relationships were observed with stress vulnerability, state anxiety, or trait anxiety.

The correlation analyses revealed consistent positive associations among stress vulnerability, cumulative life stress exposure, and both anxiety dimensions. The strongest relationship was observed between state and trait anxiety (r = 0.755, *p* < 0.001), whereas moderate correlations linked anxiety measures with stress vulnerability and cumulative life stress burden. Holmes–Rahe scores additionally showed a modest positive association with years since diagnosis, suggesting a gradual accumulation of stress burden during long-term disease management.

The scatterplot matrix provided visual confirmation of the observed correlations and demonstrated distinct clustering patterns according to autoimmune disease status. Participants with autoimmune diseases consistently exhibited higher values across the investigated psychometric dimensions, supporting the group comparisons reported in the Results section.

To further illustrate the distribution of psychometric scores across diagnostic groups, comparative boxplot analyses are presented in Figure 7.

Collectively, these findings highlight the value of computational approaches for integrated psychometric data analysis. They also demonstrate the potential of digital frameworks to support translational research, longitudinal monitoring, and personalized management strategies in autoimmune diseases.

To ensure computational transparency and reproducibility, the principal statistical indicators generated within the MATLAB framework were derived using standard formulations for correlation analysis and effect-size estimation. The strength of linear associations was quantified using the Pearson correlation coefficient (Equation (1)), whereas between-group effect sizes were estimated using Cohen’s d (Equation (2)) and the pooled standard deviation (Equation (3)).

Equation (1). Pearson correlation coefficient

(1) $$r=\frac{\sum_{i=1}^{n}(x_{i}-\bar{x})(y_{i}-\bar{y})}{\sqrt{\sum_{i=1}^{n}(x_{i}-\bar{x}{)}^{2}\cdot \sum_{i=1}^{n}(y_{i}-\bar{y}{)}^{2}}}$$

where $x_{i}$: score of participant *i* on the first variable (e.g., SVS score); $y_{i}$: score of participant i on the second variable (e.g., STAI Trait score); $\bar{x}$: mean of the first variable; $\bar{y}$: mean of the second variable; n: number of participants with complete data for both variables.

Equation (2). Cohen’s *d* effect size

(2) $$d=\frac{M_{1}-M_{2}}{s_{\mathrm{pooled}}}$$

Equation (3). Pooled standard deviation

(3) $$s_{\mathrm{pooled}}=\sqrt{\frac{(n_{1}-1)s_{1}^{2}+(n_{2}-1)s_{2}^{2}}{n_{1}+n_{2}-2}}$$

where $M_{1},M_{2}$: means of the two groups; $s_{1}^{2},s_{2}^{2}$: variances of the two groups; $n_{1},n_{2}$: sample sizes of the two groups; $s_{\mathrm{pooled}}$: a combined measure of variability from both groups.

The computational workflow implemented in MATLAB enabled reproducible data processing, automated generation of correlation matrices, multidimensional visualization, and quantitative effect-size estimation. Although the present study focused on psychometric variables, the same analytical architecture may be extended to biological, immunological, genetic, and clinical datasets. Such integration could support the future development of predictive and decision-support models in autoimmune disease research.

### 4.5. Theoretical, Clinical, and Translational Contributions

From a theoretical perspective, the present findings are consistent with biopsychosocial and psychoneuroimmunological interpretations of psychological burden in autoimmune diseases. This interpretation is consistent with the World Health Organization’s International Classification of Functioning, Disability and Health framework [68]. The framework emphasizes that health and functioning emerge from interactions among health conditions, individual characteristics, and the broader environmental and social context. Chronic stress and anxiety may interact with neuroendocrine and immune-regulatory pathways, including the hypothalamic–pituitary–adrenal axis, autonomic nervous system activity, glucocorticoid signaling, and inflammatory processes [17,18,19,20,21,60,64]. However, these biological mechanisms were not directly evaluated in the present study. The findings should therefore be interpreted as psychometric associations rather than evidence of a specific biological pathway.

The simultaneous assessment of stress vulnerability, exposure to major life events, state anxiety, and trait anxiety allowed the study to distinguish among related but conceptually different psychological dimensions. The Stress Vulnerability Scale evaluates individual susceptibility to stress in relation to lifestyle and coping resources. The Holmes–Rahe inventory quantifies exposure to significant life changes. The STAI differentiates transient anxiety from a relatively stable anxiety disposition [51,52,53,54]. The concurrent elevation of these measures in the autoimmune disease group suggests that psychological burden may involve both exposure-related and dispositional components.

A further contribution of the study is the evaluation of several complementary psychometric dimensions using the same assessment protocol in a heterogeneous autoimmune disease cohort. Previous studies have frequently focused on individual autoimmune conditions or isolated psychological outcomes [2,23,24,32,63,65]. The present findings provide exploratory evidence of a broader pattern of psychological burden within the investigated cohort. Nevertheless, because autoimmune diseases differ substantially in their clinical manifestations, severity, treatment, and prognosis, the results should not be interpreted as establishing a universal psychological profile applicable to every autoimmune disease.

From a clinical perspective, the higher stress vulnerability and anxiety scores observed in the autoimmune disease group support consideration of psychological screening within multidisciplinary care. Such screening may help identify patients who could benefit from more detailed psychological or psychiatric assessment and, when appropriate, referral to specialized mental health services [69]. The instruments used in the present study should be regarded as screening and research tools and not as stand-alone diagnostic instruments.

From a primary care and Family Medicine perspective, structured psychological assessment may facilitate earlier recognition of psychological distress and communication among primary care physicians, medical specialists, psychologists, and mental health professionals. However, the present study did not evaluate whether screening or psychological intervention improves disease activity, treatment adherence, healthcare utilization, or long-term clinical outcomes. These potential benefits require confirmation in prospective and interventional studies.

The structured electronic integration of psychometric data represents an additional translational contribution of the study. Standardized electronic data collection may facilitate consistent score calculation, data visualization, and longitudinal monitoring [70]. Nevertheless, the present study demonstrates the feasibility of structured psychometric data integration rather than the predictive performance or clinical effectiveness of a digital decision-support system. External validation and prospective evaluation are required before such a framework can be implemented for clinical prediction, risk stratification, or personalized therapeutic decision-making.

## 5. Limitations

Several limitations of the present study should be acknowledged. First, the cross-sectional design does not allow causal inferences regarding the relationship between psychological stress, anxiety, and autoimmune diseases. Longitudinal studies are needed to clarify the temporal dynamics and potential bidirectional interactions between psychological distress and immune dysregulation.

Second, autoimmune diseases were analyzed as a heterogeneous group, which limits disease-specific interpretations. This translational approach allowed the identification of shared psychological patterns associated with autoimmunity. However, autoimmune diseases differ substantially in symptom burden, disease activity, prognosis, treatment requirements, and quality-of-life impact. Consequently, the present design does not permit conclusions regarding individual autoimmune disorders. Rather, the study was intended to explore whether a common pattern of psychological distress could be identified across autoimmune conditions as a broader category. Future studies should investigate disease-specific psychological profiles and evaluate the influence of clinical severity, disease activity, treatment characteristics, and prognosis on psychological outcomes in larger, stratified cohorts.

An additional limitation relates to the recruitment strategy. Patients with autoimmune diseases were recruited from clinical settings, whereas controls were recruited from the general community. Furthermore, exclusion criteria regarding psychiatric disorders were more restrictive in the control group. These differences may have introduced selection bias and may have influenced the magnitude of the observed psychological differences between groups.

No formal a priori sample size calculation was performed. Although the final sample size allowed the detection of statistically significant group differences across the primary outcomes, future studies should incorporate prospective power analyses and more balanced recruitment strategies.

Fourth, psychological variables were assessed using self-report instruments and may therefore be influenced by subjective perception and reporting bias. In addition, disease activity was not systematically quantified using disease-specific clinical indices, and treatment regimens, socioeconomic factors, and other potential confounders were not systematically evaluated.

An additional limitation concerns the limited assessment of the social dimension. Area of residence and exposure to major life events were recorded. However, the study did not systematically assess education, employment, income, financial strain, family circumstances, social support, social isolation, stigma, healthcare access, or occupational impairment. Consequently, the social component of the biopsychosocial framework could not be directly evaluated, and residual confounding by unmeasured social factors cannot be excluded.

Finally, the marked predominance of female participants reflects the epidemiology of autoimmune diseases but may have influenced psychological outcomes, given known sex-related differences in stress perception and anxiety. In addition, the autoimmune and control groups differed with respect to sex distribution and age structure. Although these differences reflect the characteristics of the recruited populations, adjusted multivariable analyses accounting for the potential confounding effects of age and sex were not performed and should be incorporated in future investigations.

## 6. Future Directions

Future studies should adopt integrative multidisciplinary approaches combining psychometric, clinical, immunological, genetic, and molecular data [71,72,73]. The incorporation of objective biomarkers, including inflammatory cytokines, stress-related hormones, genetic susceptibility markers, and immune-cell profiling, may provide a more comprehensive understanding of the psychoneuroimmunological mechanisms involved in autoimmune diseases.

Consistent with a biopsychosocial approach, future studies should simultaneously assess biological, psychological, and social determinants [73]. Future research should assess disease activity and relevant biomarkers alongside key social determinants. These should include education, employment, financial strain, healthcare access, family burden, social support, social isolation, stigma, and participation in occupational and community activities. Recent disease-specific evidence suggests that emotional, informational, and social-interaction support may be associated with disease activity in systemic lupus erythematosus [74]. However, the observational design of the available evidence does not permit causal conclusions. Longitudinal and disease-specific studies are needed to clarify the temporal and potentially bidirectional relationships among autoimmune disease, exposure to major life events, stress vulnerability, anxiety, and social context.

The integration of multidimensional datasets within structured electronic health record systems and digitally assisted analytical platforms may facilitate improved risk stratification, longitudinal monitoring, and personalized management strategies. Such approaches may further support the development of predictive computational models and decision-support systems for translational psychoneuroimmunology research [75].

The convergent reporting framework proposed in the present study may reduce limitations associated with self-reported data by integrating objective clinical and paraclinical information within unified digital infrastructures. This approach may be particularly relevant in primary care, family medicine, and oral medicine, where extensive clinical networks facilitate early recognition of immune-mediated disorders.

Several autoimmune and immune-mediated conditions, including Sjögren syndrome, pemphigus vulgaris, oral lichen planus, and oral manifestations of systemic lupus erythematosus, may initially present within dental practice [76]. Consequently, dedicated convergent EHR-based approaches for dentistry and oral medicine may represent a promising area for future investigation and implementation [77].

Despite the limitations of the present study, the findings provide a foundation for future research on psychoneuroimmunological mechanisms underlying autoimmunity. They also support the continued development of integrated computational infrastructures for clinical monitoring, data analysis, and personalized patient management.

## 7. Conclusions

This study showed that patients with autoimmune diseases exhibited significantly higher levels of stress vulnerability, cumulative life stress exposure, and both state and trait anxiety compared with healthy controls. From a translational perspective, the findings highlight psychological distress as a shared and clinically relevant feature across autoimmune conditions rather than a disease-specific phenomenon.

Notably, the pronounced elevation of trait anxiety suggests that anxiety may represent a stable psychological characteristic associated with autoimmune disease status. This finding supports its integration within a psychoneuroimmunological framework of autoimmunity. In contrast, no association was observed with the Type A psycho-behavioral pattern. This finding suggests that the observed psychological burden is unlikely to reflect fixed personality traits and may instead involve disease-related and potentially modifiable processes.

Together, these findings underscore the importance of incorporating psychological assessment and stress-management strategies into comprehensive autoimmune disease care. Addressing psychological distress alongside immunological and clinical factors may contribute to improved patient well-being and support more integrated and personalized therapeutic approaches in autoimmune disorders.

The use of complementary statistical and computational analytical approaches strengthened the robustness and reproducibility of the findings. Furthermore, the MATLAB-based framework demonstrated the feasibility of integrating psychometric data within a structured digital environment, enabling multidimensional visualization, correlation mapping, and reproducible computational analysis.

Finally, the proposed computational framework highlights the potential of digitally assisted approaches for the integration of psychological, clinical, and biological information. Such systems may facilitate longitudinal monitoring, personalized patient management, predictive modeling, and future translational research aimed at elucidating the complex interactions between psychological distress and autoimmune disease.

## Figures and Tables

**Table 1:** Distribution of autoimmune diseases in the study cohort (n = 204).

Disease	*n* (%)
Psoriasis	59 (28.9)
Autoimmune thyroiditis	51 (25.0)
Oral lichen planus	30 (14.7)
Rheumatoid arthritis	14 (6.9)
Other autoimmune diseases	14 (6.9)
Type 1 diabetes mellitus	12 (5.9)
Inflammatory bowel disease	11 (5.4)
Ankylosing spondylitis	5 (2.5)
Pemphigus vulgaris with oral involvement	3 (1.5)
Systemic lupus erythematosus	2 (1.0)
Multiple sclerosis	2 (1.0)
Myasthenia gravis	1 (0.5)
Total	204 (100.0)

Data are presented as number of participants (*n*) and percentage (%).

**Table 2:** Sociodemographic characteristics of the study population.

Variable	Autoimmune Patients (*n* = 204)	Controls (*n* = 185)
Female sex, *n* (%)	180 (88.2)	128 (69.2)
Male sex, *n* (%)	24 (11.8)	57 (30.8)
Urban residence, *n* (%)	163 (80.0)	165 (89.2)
Rural residence, *n* (%)	41 (20.0)	20 (10.8)

Data are presented as number of participants (*n*) and percentage (%).

**Table 3:** Vulnerability to stress scores in patients with autoimmune diseases and healthy controls.

Group	Mean ± SD	Mean Difference (95% CI)	*p*-Value
Autoimmune patients	26.31 ± 14.51	5.31 (2.59–8.03)	<0.001
Controls	21.00 ± 12.58	—	—

Data are presented as mean ± standard deviation (SD). Mean difference and 95% confidence interval (CI) were calculated using an independent samples *t*-test.

**Table 4:** Comparison of Holmes–Rahe Stress Scale scores between patients with autoimmune diseases and healthy controls.

Group	Mean ± SD	Mean Difference (95% CI)	*p*-Value
Autoimmune patients	339.25 ± 197.89	101.73 (64.37–139.09)	<0.001
Controls	237.51 ± 174.54	—	—

Data are presented as mean ± standard deviation (SD). Mean differences with 95% confidence intervals (CI) and *p*-values were obtained using an independent samples *t*-test.

**Table 5:** Comparison of state and trait anxiety scores (STAI-1 and STAI-2) between patients with autoimmune diseases and healthy controls.

Scale	Autoimmune Patients, Mean ± SD	Controls, Mean ± SD	Mean Difference (95% CI)	*p*-Value
STAI-State	43.46 ± 12.62	36.91 ± 12.32	6.55 (4.06–9.04)	<0.001
STAI-Trait	46.95 ± 10.29	39.49 ± 11.76	7.46 (5.25–9.67)	<0.001

Data are presented as mean ± standard deviation (SD). The mean difference is reported with its 95% confidence interval (CI). The independent-samples *t*-test was used for STAI-State, while Welch’s *t*-test was used for STAI-Trait because the homogeneity-of-variance assumption was not satisfied.

**Table 6:** Integrated comparison of psychometric outcomes according to autoimmune disease status.

Measure	Autoimmune Patients (*n* = 204), Mean ± SD	Controls (*n* = 185), Mean ± SD	t (df)	*p*-Value	Cohen’s d
SVS Vulnerability	26.31 ± 14.51	21.00 ± 12.58	3.836 (387)	<0.001	0.390
STAI State Anxiety	43.46 ± 12.62	36.91 ± 12.32	5.172 (387)	<0.001	0.525
STAI Trait Anxiety	46.95 ± 10.29	39.49 ± 11.76	6.624 (367.6)	<0.001	0.677
Holmes–Rahe LCU	339.25 ± 197.89	237.51 ± 174.54	5.354 (387)	<0.001	0.544

Data are presented as mean ± standard deviation (SD). Between-group comparisons were performed using independent-samples *t*-tests. Welch’s *t*-test was used for STAI Trait Anxiety because the homogeneity-of-variance assumption was not satisfied; therefore, adjusted degrees of freedom are reported. Effect sizes were expressed as Cohen’s d. All tests were two-tailed.

**Table 7:** Pearson correlation matrix among psychometric variables, age, and disease duration.

Measure	SVS Vuln	STAI State	STAI Trait	Holmes-Rahe	Age	Years Since Dx
Stress Vulnerability Scale score	—	0.352 ***	0.437 ***	0.169 ***	−0.007	−0.030
State Anxiety Inventory (STAI-1)	0.352 ***	—	0.755 ***	0.267 ***	−0.075	0.017
Trait Anxiety Inventory (STAI-2)	0.437 ***	0.755 ***	—	0.296 ***	−0.078	−0.006
Holmes–Rahe Life Stress Inventory score	0.169 ***	0.267 ***	0.296 ***	—	0.217 ***	0.207 **
Age	−0.007	−0.075	−0.078	0.217 ***	—	0.284 ***
Years since Dx	−0.030	0.017	−0.006	0.207 **	0.284 ***	—

Note. ** *p* < 0.01; *** *p* < 0.001. Abbreviations: Years since Dx, years since autoimmune disease diagnosis.

## Data Availability

The aggregated data supporting the findings of this study are presented in the article and Supplementary Materials. Participant-level clinical and psychometric data are not publicly available because the conditions of informed consent and ethics approval did not provide for unrestricted public sharing of potentially sensitive information. Further requests for additional aggregated or deidentified information may be directed to the corresponding authors and will be considered in accordance with applicable ethical, institutional, and data-protection requirements.

## Supplementary Materials

The following supporting information can be downloaded at https://www.mdpi.com/article/10.3390/healthcare14162584/s1. Figure S1. Distribution of the Type A psycho-behavioral pattern in patients with autoimmune diseases and healthy controls; Table S1. Descriptive statistics for psychometric measures in the overall study population; Table S2. Distribution of participants according to psychometric score categories by autoimmune disease status.

## Abbreviations

The following abbreviations are used in this manuscript:

ADs	Autoimmune diseases
CRP	C-reactive protein
HPA	Hypothalamic–pituitary–adrenal
IL	Interleukin
JAS	Jenkins Activity Survey
MS	Multiple sclerosis
MATLAB	The MATLAB environment is a highly configurable, interactive platform designed for technical computing, data analysis, visualization, and algorithm development.
PNI	Psychoneuroimmunology
RA	Rheumatoid arthritis
SD	Standard deviation
SLE	Systemic lupus erythematosus
STAI	State–Trait Anxiety Inventory
STAI-1	State Anxiety subscale of the State–Trait Anxiety Inventory
STAI-2	Trait Anxiety subscale of the State–Trait Anxiety Inventory
SVS	Stress Vulnerability Scale
TABP	Type A behavior pattern
TNF-α	Tumor necrosis factor alpha
WHO	World Health Organization
