# Supplementary material for: Psychological Stress and Anxiety in Autoimmune Diseases: A Cross-Sectional Case–Control Study
Source: Healthcare (Basel). 2026 Aug 17;14(16):2584. doi: 10.3390/healthcare14162584 (PMC13511814; doi:10.3390/healthcare14162584)
Supplement: Supplementary file 1 [file healthcare-14-02584-s001.zip › healthcare-4447806-supplementary.pdf]

Supplementary Materials

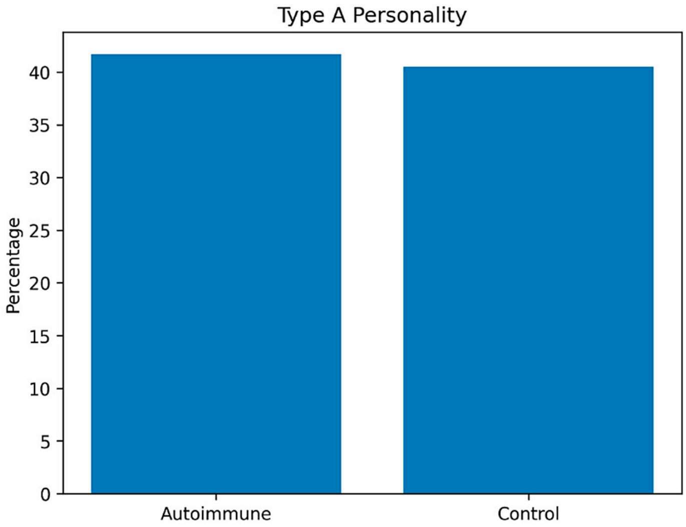

Figure S1. Distribution of the Type A psycho-behavioral pattern in patients with autoimmune diseases and healthy controls. Data are presented as percentages of participants within each group.

Supplementary Table S1. Descriptive statistics for psychometric measures in the overall study population (N = 389).

Measure	Mean	SD	Median	Range
SVS Vulnerability	23.68	13.82	22.0	0–79
STAI State Anxiety	42.39	10.21	41.0	24–71
STAI Trait Anxiety	44.86	9.40	45.0	26–70
Holmes–Rahe LCU	332.51	217.38	294.0	0–1121

Note. Data are presented as mean, standard deviation (SD), median, and range.

Supplementary Table S2. Distribution of participants according to psychometric score categories by autoimmune disease status.

Diagnosis	SVS Vulnerability (Low/Moderate/High)	STAI State Anxiety (Low/Moderate/High)	STAI Trait Anxiety (Low/Moderate/High)	Holmes–Rahe (Low/Moderate/High)
No (n = 185)	141 / 40 / 4	99 / 50 / 36	79 / 62 / 44	56 / 62 / 67
Yes (n = 204)	127 / 65 / 12	53 / 83 / 68	27 / 84 / 93	30 / 49 / 125

Legend: Participants were classified into low, moderate, and high categories according to instrument-specific cut-off values. Values represent the number of participants within each category.
